# Comparative Performance Analysis of Inverse Phase Active Vibration Cancellation Using Macro Fiber Composite (MFC) and Vibration Absorption of Silicone Gel for Vibration Reduction

**DOI:** 10.3390/polym15244672

**Published:** 2023-12-11

**Authors:** Sang-Un Kim, Joo-Yong Kim

**Affiliations:** 1 Department of Smart Wearable Engineering, Soongsil University, Seoul 06978, Republic of Korea; tkddnsl0723@naver.com; 2 Department of Materials Science and Engineering, Soongsil University, Seoul 06978, Republic of Korea

**Keywords:** macro fiber composite, active vibration cancellation, vibration control, piezoelectric composite material, passive gel

## Abstract

This study focuses on addressing the issue of unwanted vibrations commonly encountered in various fields by designing an Active Vibration Cancellation (AVC) structure using a flexible piezoelectric composite material macro fiber composite (MFC). A comparative performance analysis was conducted between the AVC and a traditional passive gel that continuously absorbs vibrations. The results showed that AVC was more effective in mitigating vibrations, making it a promising solution for vibration control. The results of this study from extensive vibration–sensing experiments and comparisons revealed that AVC effectively cancels the vibrations and vibration absorption performance of the passive gel. These findings underline the potential of AVC as an efficient method for eliminating and managing undesired vibrations in practical applications. Specifically, AVC demonstrated a high vibration cancellation ratio of approximately 0.96 at frequencies above 10 Hz. In contrast, passive gel exhibited a relatively consistent vibration absorption ratio, approximately 0.70 to 0.75 at all tested frequencies. These quantitative findings emphasize the superior performance of AVC in reducing vibrations to levels below a certain threshold, demonstrating its efficacy for vibration control in real-world scenarios.

## 1. Introduction

Smart materials, particularly piezoelectric materials, are increasingly being studied in the field of vibration control. Piezoelectric materials can convert the deformation caused by vibrations into an electrical signal that can be used for self-sensing. Conversely, deformation can be induced by applying an electrical signal, utilizing the inverse piezoelectric effect. However, traditional piezoelectric materials, such as ceramic-based lead zirconate titanate (PZT), have limited flexibility and cannot be easily shaped to conform to the surface of a vibrating structure. Additionally, their deformation capabilities are limited, which restricts their effectiveness in vibration–control applications. In response to these limitations, new piezoelectric materials, such as PZT fiber used in MFC [1,2], have been developed. These materials provide greater flexibility [3] and displacement capabilities [4,5], making them better suited for vibration–control applications [6,7].

The MFC is a revolutionary composite actuator that was recently developed at the NASA Langley Research Center [1]. An MFC consists of piezoelectric fibers embedded in a flexible polymer matrix. Due to their composite structure, they have the flexibility to conform to curved surfaces, making them suitable for vibration control and structural health–monitoring applications [8]. MFC offers several advantages over traditional piezoelectric materials, including a higher strain output, greater flexibility, and easier integration into structures [9,10]. As a result, MFC is an attractive option for a wide range of applications, including aerospace [2,11,12], civil engineering [13,14], and other fields where vibration control [15] and sensing are critical.

Various vibration control methods have been developed including passive damping [16,17], active control [18,19], and semi-active control [20]. Passive damping involves optimizing the properties and shape of damping materials to absorb energy and reduce the amplitude of vibrations according to the attachment surface, location, shape, and intensity of the vibrations. Typical materials suitable for this include rubber [21,22,23], silicone [24,25,26], and viscoelastic polymers [27,28,29]. They are used to reduce basic vibrations due to their low cost, ease of production, and processability. In one study, vibrations were passively filtered by using physical bonds within the gel structure to separate and recombine the vibrations above a certain frequency [30]. Another study confirmed that PVA gel, which has chemical and physical network bonds within a single polymer, efficiently absorbs vibrations over a wide frequency range [31].

However, the viscoelastic properties of polymer gels are significantly affected by the nature and structure of their bonds. In the case of hydrogels, these properties are further affected by moisture content. Moreover, external environmental temperature variations and the target vibration frequency can break the bonds and alter the viscoelastic properties. Therefore, vibrational absorption by polymer gels has several limitations.

In contrast, active vibration control involves the use of actuators to apply force to the structure to counteract vibrations. Numerous publications have reviewed the applications of smart materials. In an experiment to control the vibration of a cantilever beam using an MFC actuator, a study was conducted to reduce vibration at a frequency of 26.6 Hz by optimizing the applied voltage [13]. One study demonstrated the potential to suppress vibrations by attaching MFC actuators to a curved surface and successfully suppressing vibrations with a frequency of 12.8 Hz [32].

In this study, referring to the study, we modeled a textile actuator with active vibration cancellation capability using MFC. We also compared and analyzed the performance with a passive damping material, silicone gel, to confirm the suitability and potential of the vibration cancellation method. The vibrations to be canceled were generated in the frequency range of 1 to 50 Hz using an MFC vibrator, and the voltage was measured through the piezoelectric effect of the MFC.

## 2. Materials and Methods 

### 2.1. MFC d31 Type and d33 Type

MFC exhibits distinct structures that can be categorized into two primary types: the d31 and d33 types. These classifications pertain to the orientation of the embedded piezoelectric fibers within the composite structure, which dictates their specific piezoelectric behaviors.

The d31 type of MFC was meticulously engineered to contract when subjected to an electric field applied perpendicular to its surface, polarizing in the thickness direction. This unique polarization pattern induces a characteristic known as the d31 piezoelectric coefficient. The d31 coefficient quantifies the material response in generating an electric charge when experiencing a mechanical strain or deformation perpendicular to its surface. The orientation of the piezoelectric fibers within the composite structure enabled the d31-type MFC to efficiently convert electrical energy into mechanical energy along the thickness direction.

In contrast to the d31 variant, the d33 type of MFC was engineered with a different orientation of the piezoelectric fibers. This design was optimized to expand or contract parallel to the surface upon exposure to an electric field applied in the same direction. This polarization induces the d33 piezoelectric coefficient, which quantifies the material’s response in generating an electric charge when subjected to mechanical strain or deformation along the plane of its surface. 

**Figure 1:**
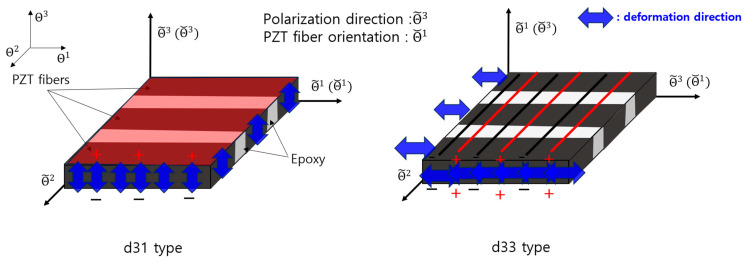
Schematic of 3 coordinate systems for d31 type and d33 type.

This paper used three coordinate systems: the curvilinear coordinate system, the fiber coordinate system, and the polarization coordinate system. Ref. [33], respectively, by $\Theta^{i},\;{\overset{˘}{\Theta }}^{i},\;{\tilde{\Theta }}^{i}\;\left(i=1,\;2,\;3\right),$ as shown in Figure 1.

In geometry, the curvilinear coordinate system is applied to represent the three-axis directions, the thickness direction, and the other two axes denoting the in-plane directions for the MFC. The fiber coordinate system defines the fiber orientations represented by ${\overset{˘}{\Theta }}^{1}$. The polarization coordinate system defines the piezoelectric effects of the MFC, specifically the d31 and 33 types. In summary, with the three types of coordinate systems, we can understand the PZT fiber orientations using the fiber coordinate system and the directions of the piezoelectric effects using the polarization coordinate system for both types of the MFC. In this study, to achieve active vibration cancellation, we utilized a d31 type of MFC that vibrates in the thickness direction, effectively creating inverse-phase vibrations opposite to the direction of the original vibration.

In this study, we used MFC P2 type (d31 type) films purchased from Smart Material Corp. The engineering characteristics associated with it are summarized in Table 1, which presents the properties provided by the Smart Material Corp [2]. The piezoelectric effect and inverse piezoelectric effect of the MFC actuator can be represented by the constitutive equations as shown in Equations (1) and (2) [33]. Deformation due to vibrations in the MFC generates a sensing voltage, whereas the application of a voltage deforms the MFC.

(1) $$\overset{˘}{\varepsilon }=\overset{˘}{s}\overset{˘}{\sigma }+\overset{˘}{d}\overset{˘}{E}$$

(2) $$\overset{˘}{D}=\overset{˘}{d}\overset{˘}{\sigma }+\overset{˘}{\epsilon }\overset{˘}{E}$$

### 2.2. Active Vibration Cancellation (AVC)

**Figure 2:**
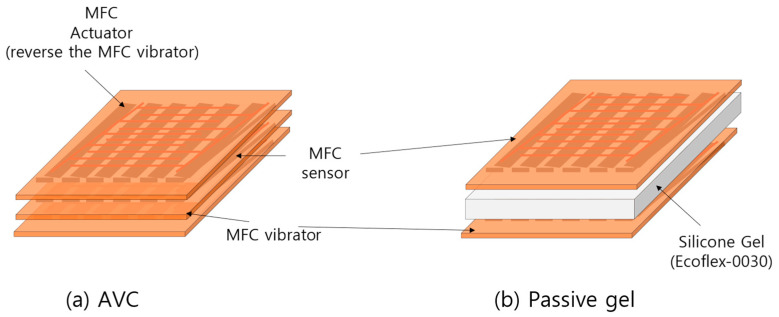
Structures of (**a**) AVC and (**b**) Passive gel.

As shown in Figure 2a, the AVC structure with MFCs was designed to generate vibrations in the inverse phase with the generated vibrations. The bottom layer served as the MFC vibrator capable of generating vibrations to cancel out unwanted vibrations, whereas the middle layer housed the sensor for detecting vibrations. The top layer contained the MFC actuator, which generated inverse–phase vibrations by reversing the MFC vibrator of the AVC. Additionally, we used an adhesive layer between each layer to ensure that there were no gaps.

For a performance comparison, the passive gel structure, as depicted in Figure 2b, was designed to minimize any structural performance differences. The MFC vibrator and MFC sensor were positioned around the passive gel to match their dimensions. The passive gel used in this setup was produced with Smooth-On Inc. (Macungie, PA, USA)’s silicone rubber Ecoflex-0030, with a thickness of 1.0 mm, matching the width of the MFC, to be used for vibration damping.

**Figure 3:**
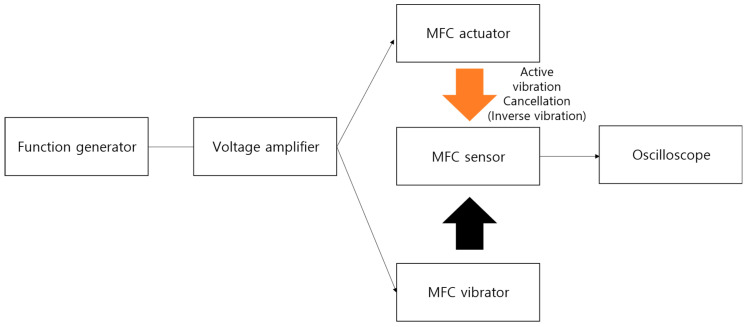
System block diagram of AVC using MFC sensor and MFC actuator.

For the AVC experiments, as shown in Figure 3, we used the Analog Discovery 2 Digilent Inc. (Pullman, WA, USA), which incorporates an oscilloscope and function generator functionalities. The MFC actuator and MFC vibrator were provided with voltage amplification within the peak-to-peak voltage range of −60 to 340 Vpp, falling within their operating voltage limits. For the MFC sensor, we conducted sensing with an oscilloscope at a sampling frequency of 800 Hz for a duration of 10 s to evaluate the performance of the vibration, AVC, and the passive gel.

## 3. Results

### 3.1. Vibration Sensing of no AVC

**Figure 4:**
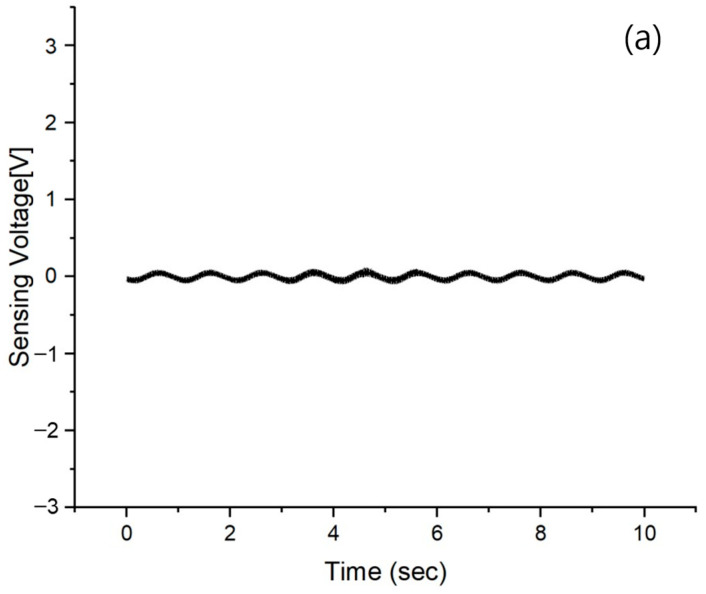

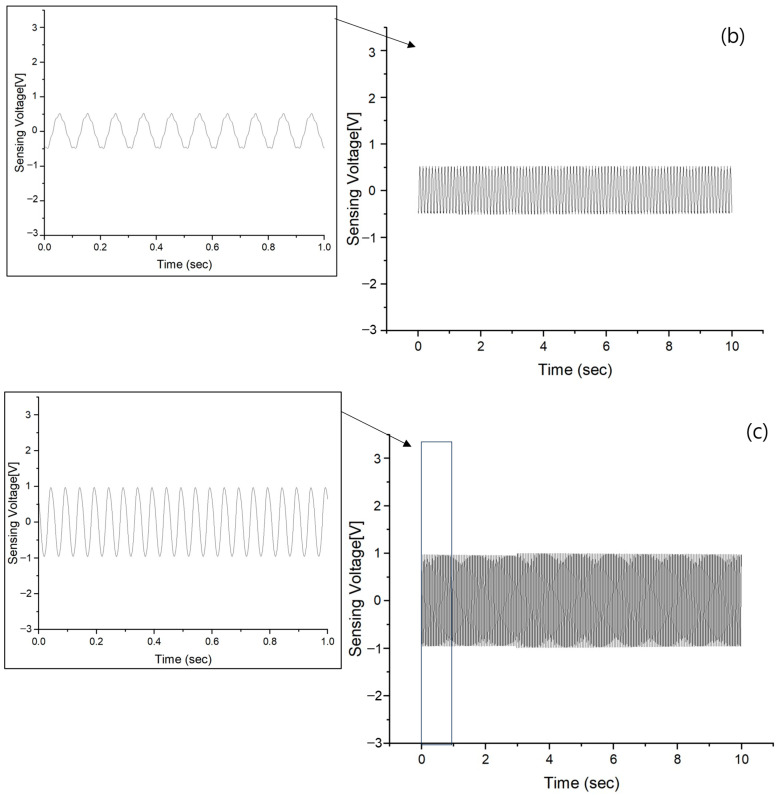

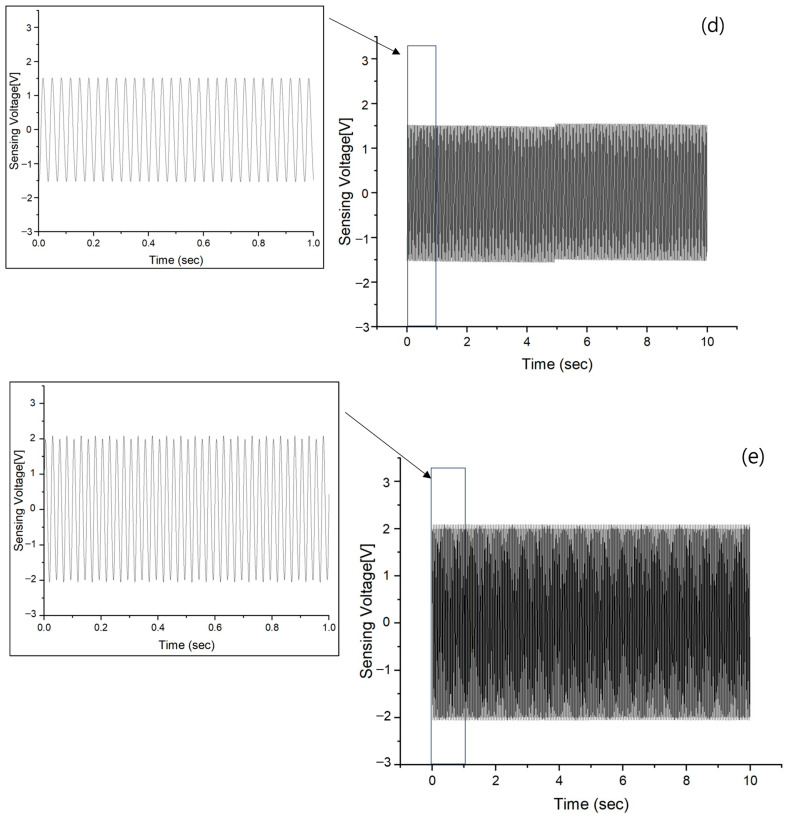

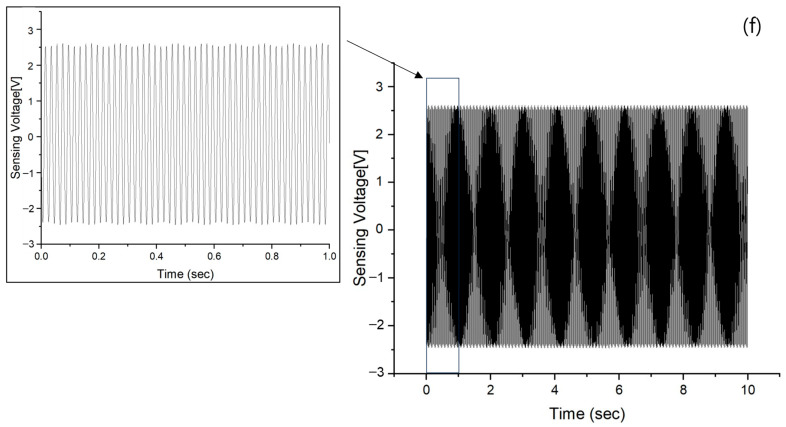
MFC sensing voltage of No AVC at different frequencies (**a**) 1 Hz, (**b**) 10 Hz, (**c**) 20 Hz, (**d**) 30 Hz, (**e**) 40 Hz, and (**f**) 50 Hz.

**Figure 5:**
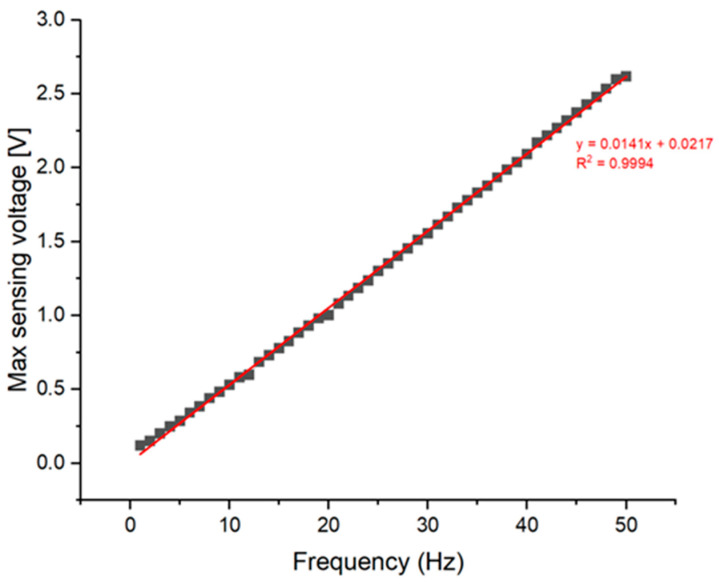
Max sensing voltage of No AVC at frequency range 1 to 50 Hz.

To assess the performance of the AVC, we initially conducted measurements solely on the vibrations produced by the MFC vibrator, excluding the operation of the MFC actuator. The results are shown at 10 Hz intervals within the 1 to 50 Hz frequency range, as shown in Figure 4. The voltage measurements exhibited varying ranges: (a) ranged from −0.1 to 0.12 V, (b) from −0.5 to 0.53 V, (c) from −0.98 to 1.0 V, and (d) from −1.56 to 1.55 V, (e) from −2.05 to 2.09 V, and (f) from −2.46 to 2.61 V. with the range of between the lowest and highest voltage. Notably, there was an observable trend of increasing sensing voltage at higher frequencies. Furthermore, it is worth mentioning that each vibration measurement at a different frequency corresponds precisely to the applied frequency. Figure 5 shows a graphical representation of the maximum sensing voltage as a function of frequency, encompassing measurements across the 1 to 50 Hz spectrum. This graph illustrates a high degree of linearity, characterized by a coefficient of determination of 0.9994. 

Upon examining the trend of the sensing voltage in Figure 4, it is evident that there is an absence of harmonic oscillations attributable to external influences. This observation signifies a notable absence of resonance vibrations induced by external factors within the measured sensing voltage data. The discerned absence of harmonic vibrations within the recorded sensing voltage traces is indicative of a system that is remarkably immune to external perturbations. Furthermore, the absence of discernible harmonic patterns within the sensing voltage trend underscores the effectiveness of the implemented isolation and damping mechanisms.

As shown in Figure 5, the maximum sensing voltage consistently increased with the frequency, indicating the absence of resonance within the measured frequency range. The consistent increase in the maximum sensing voltage with respect to frequency across the observed range signifies a lack of resonance occurrences. This absence of resonance points towards the system’s capability to maintain stability without succumbing to resonance-induced fluctuations within the specified frequency range.

### 3.2. Vibration Sensing of Passive Gel

**Figure 6:**
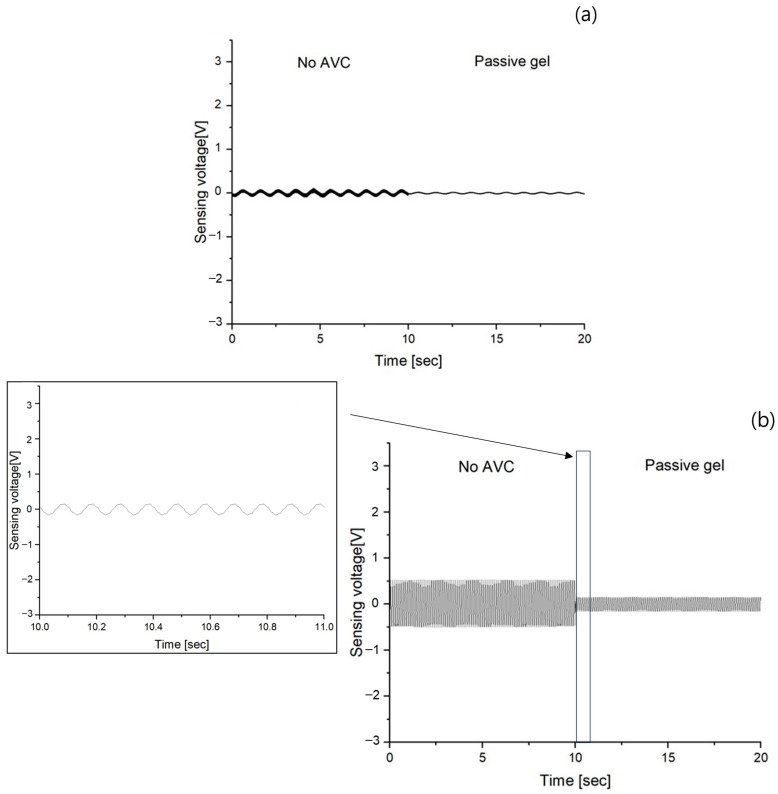

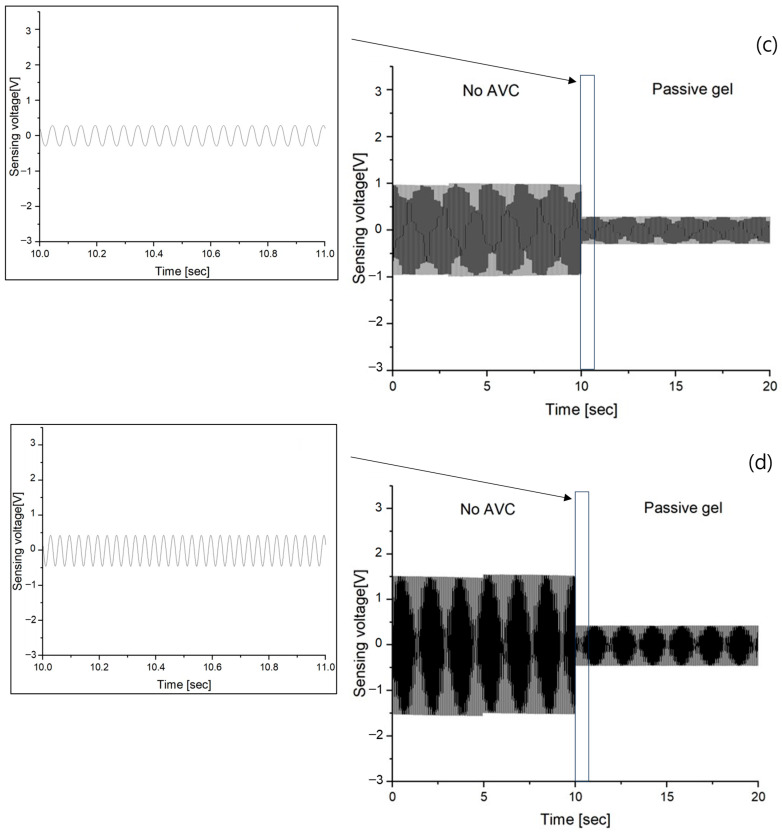

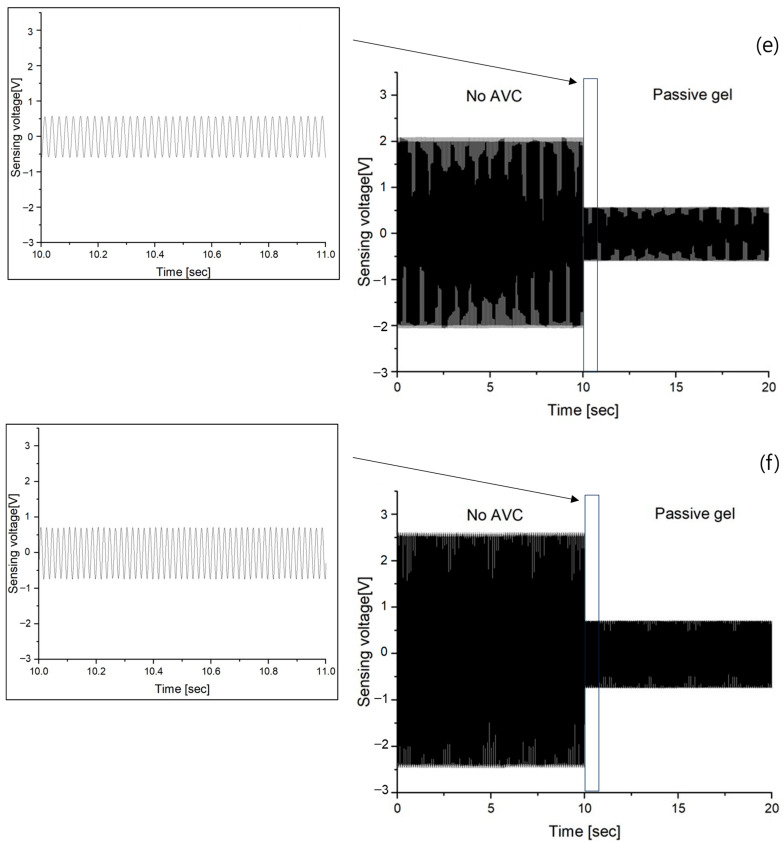
MFC sensing voltage of No AVC (0~10 s) and passive gel (10~20 s) at different frequencies (**a**) 1 Hz, (**b**) 10 Hz, (**c**) 20 Hz, (**d**) 30 Hz, (**e**) 40 Hz, and (**f**) 50 Hz.

In order to clearly confirm the vibration of no AVC and the vibration through passive gel, two vibration graphs measured for 10 s were shown in continuous time as shown in Figure 6. The range from 0 to 10 s was the result of no AVC vibration, and the range from 10 to 20 s was the result of passive gel vibration. Vibrations of the same frequency were confirmed. The results are (a) ranged from −0.029 to 0.029 V, (b) from −0.16 to 0.16 V, (c) from −0.3 to 0.3 V, (d) from −0.45 to 0.43 V, (e) from −0.6 to 0.6 V, and (f) from −0.75 to 0.71 V. In the vibration sensing voltage graph, due to the lack of precise vibration visibility within the 10~50 Hz range, an enlarged graph of the data from 10 to 11 s was inserted. This was implemented to facilitate frequency confirmation and enable clearer visualization of the amplitude variations.

### 3.3. Vibration Sensing of AVC

**Figure 7:**
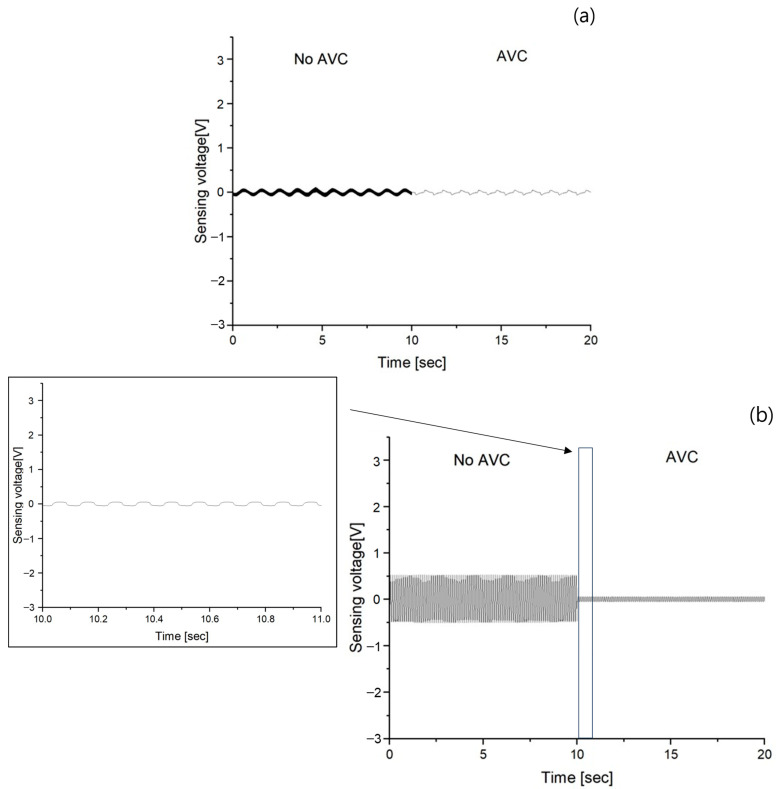

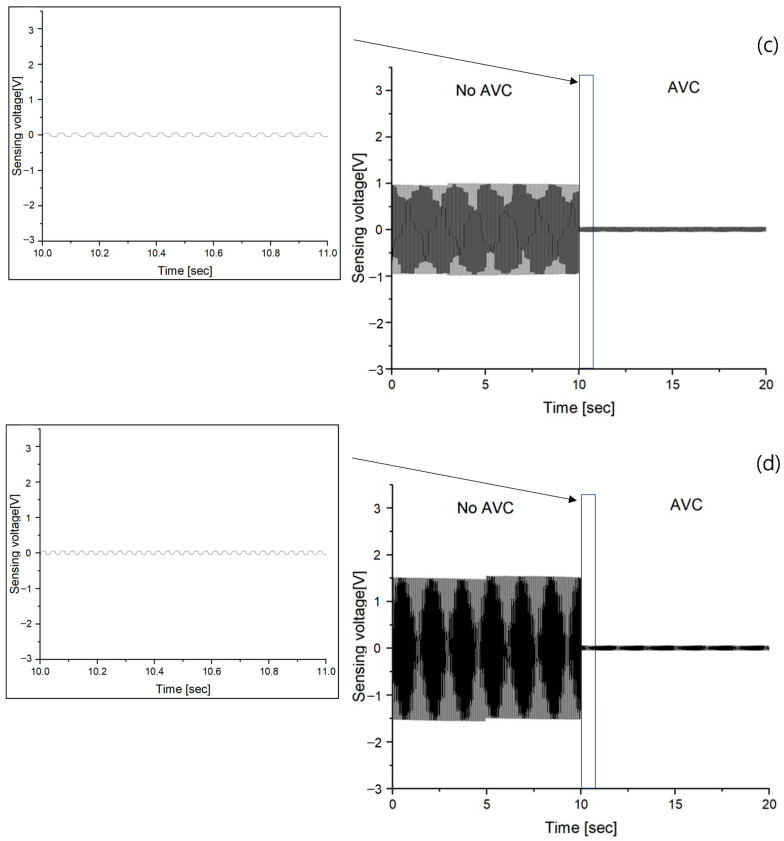

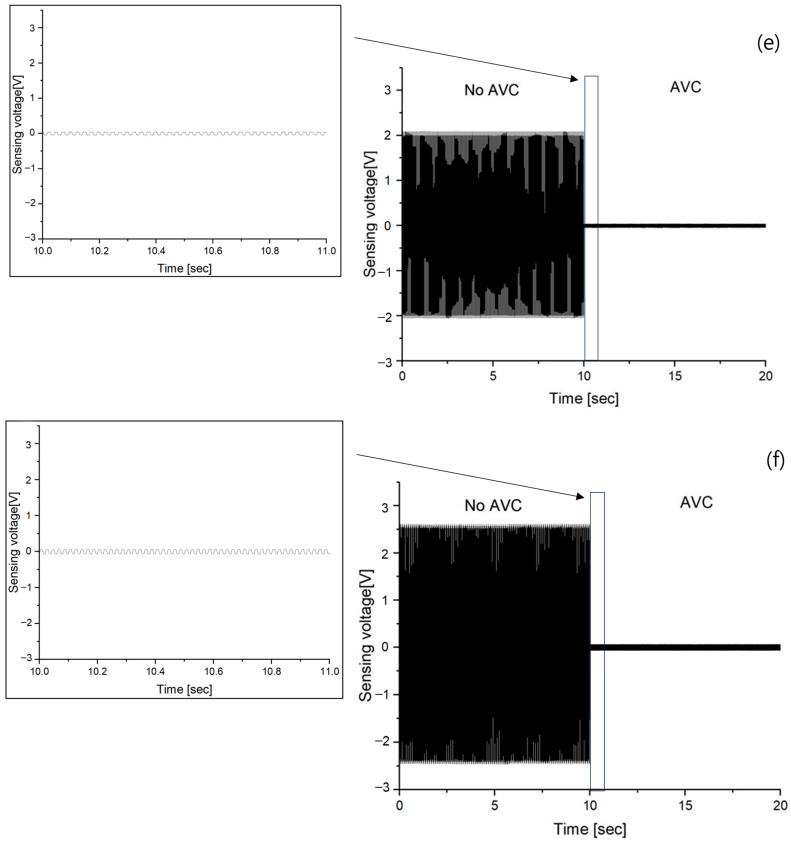
MFC sensing voltage of No AVC (0~10 s) and AVC (10~20 s) at different frequency (**a**) 1 Hz, (**b**) 10 Hz, (**c**) 20 Hz (**d**) 30 Hz, (**e**) 40 Hz, and (**f**) 50 Hz.

In the case of the passive gel, the vibration–sensing voltage after vibration absorption is shown in Figure 7 in the same manner as the AVC result. The results of AVC are (a) ranged from −0.058~0.049 V, (b) from −0.05~0.057 V, (c) from −0.048~0.062 V, (d) from −0.045~0.063 V, (e) from −0.049~0.04 V, and (f) from −0.068~0.064 V. Similar to the passive gel sensing voltage graph, the 10~50 Hz range lacked precise visibility of vibrations, prompting the insertion of an enlarged graph displaying data from 10 to 11 s. This implementation was aimed at facilitating easier frequency confirmation and providing a clearer visualization of amplitude variations.

**Figure 8:**
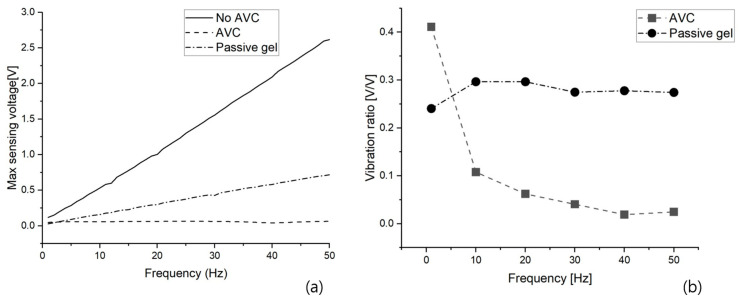
Comparison of Vibrations by Frequency: (**a**) Max Sensing Voltage, and (**b**) Vibration Ratio.

All the vibration results are shown by the frequency based on the maximum sensing voltage as shown in Figure 8a. To compare the performance of AVC and the vibration absorption of the passive gel, the results are expressed as a ratio to no AVC based on the maximum sensing voltage using Equation (3). This is shown in Figure 8b and Table 2.

Vibration ratio = Max sensing voltage (AVC, passive gel)/max sensing voltage of no AVC (3)

## 4. Discussion

The vibrations of no AVC were sensed using only the MFC vibrator, without engaging the MFC actuator. These measurements exhibited consistent maximum and minimum sensing voltages without any notable noise or complex frequency components. Additionally, the results of the frequency of the vibrations depending on the time were found to match the excitation frequencies precisely. As a result, it was established that the vibrations with no AVC are suitable for evaluating the vibration absorption performance of AVC and passive gel.

A comparison of the performance of the AVC and passive gel, which was the main focus of this study, first confirmed that both structures effectively cancel and absorb vibrations. Regarding the vibration absorption by passive gel, as shown in Figure 6, it was observed that vibrations within the 1 to 50 Hz frequency range, with 10 Hz intervals as in the case of no AVC vibrations, were absorbed, resulting in a significant reduction in the maximum sensing voltage. However, similar to the increase in the maximum sensing voltage observed in the absence of AVC vibrations with increasing frequency, the maximum sensing voltage increased with frequency even in the presence of passive gel vibration absorption. However, for AVC, which cancels vibrations through inverse-phase vibration, as shown in Figure 7, the maximum sensing voltages of AVC vibrations did not vary significantly with frequency. To further analyze the variation in the maximum sensing voltage between the no–AVC and AVC cases, Figure 8a was examined. Both the no–AVC and the passive gel exhibited a gradual increase in the maximum sensing voltage with frequency, while the maximum sensing voltage for AVC remained consistently below approximately 0.065 V, regardless of the frequency. For a more quantitative comparison of the relative vibration cancellation performance against the frequency for no–AVC, the results are represented in Figure 8b and Table 2. AVC demonstrated a high vibration cancellation ratio of approximately 0.59 at 1 Hz and exceeding 0.96 at frequencies above 10 Hz. In contrast, the passive gel exhibited a relatively consistent vibration absorption ratio of approximately 0.70~0.75 at all frequencies.

The difference in the vibration ratio between these two structures was attributed to the method of absorbing vibration energy in the passive gel, which absorbs vibrational energy within its molecular structure, and the method of generating inverse phase vibrations to cancel out the existing vibrational energy in the AVC. Ultimately, in terms of performance, it was determined that AVC, which can effectively cancel out undesired vibrations to a level below a certain threshold under the conditions of this study, outperforms passive gel, which maintains a consistent level of vibration absorption. Table 3 presents a comparative analysis of the effectiveness with other studies aimed at mitigating vibrations. In a study using two identical MFC actuators to dampen structural vibration, the initial vibration was reduced to 0.3. Utilizing quasi-zero-stiffness isolators, the vibration was further decreased to 0.22 compared to both the existing device and thin layer composite [34]. With the application of an unimorph piezoelectric driver and sensor (THUNDER) actuator for vibration isolation, the vibration was reduced to 0.37 of its initial level [35]. This study effectively demonstrated superior preoperative vibration reduction compared to studies focusing on vibration suppression or isolation.

The method employed in this paper to generate inverse–phase vibrations, known as the MFC actuator, involves reversing the same MFC used for the MFC vibrator without the need for an additional control system. In addition to vibration cancellation research on MFC, much research is being conducted on systems that ultimately control vibration. As a control system, there has also been research on active controllers using the Linear Quadratic Gaussian (LQG) theory [36] and research on vibration as a control system using a feedforward strategy [37].

In research on control systems, it is important to minimize the response time of the signal system and increase the vibration reduction effect for non-periodic random signals. Consequently, it is anticipated that there might have been a slight delay in the AVC operation. In the case of the actual application of AVC, the structural part must be determined to calculate the delay for vibration to enable accurate antiphase vibration cancellation of the AVC. However, with proper signal processing and optimization, we believe that this can lead to the development of an even higher–performing AVC.

## 5. Conclusions

This study developed an Active Vibration Cancellation (AVC) structure using a flexible piezoelectric composite material, MFC, to address and manage unwanted vibrations prevalent across various industries. To evaluate its performance, we conducted comparative experiments with a traditional silicone–based passive gel that is commonly used for continuous vibration absorption.

The results presented clear evidence supporting the AVC system. This distinctly demonstrated AVC’s superior suitability for vibration cancellation, demonstrating its notably enhanced efficacy in vibration mitigation compared to passive gels. These robust experimental findings underscore the significance of MFC technology in the realm of vibration control and present a far more effective solution than conventional passive gels. This emphasizes the potential for employing AVC structures in diverse fields that require precise vibration control.

Furthermore, future research will focus on advancing vibration control systems, aiming for an even greater enhancement of the cancellation effects. This ongoing effort seeks to refine the existing AVC system and explore methods utilizing new materials or technologies to further augment cancellation effectiveness, ultimately striving for superior performance in vibration–control systems.

## Figures and Tables

**Table 1:** The Engineering properties of MFC d31 type.

Engineering Properties	Symbol	MFC d31 Type [2]
Young’s modulus [GPa]	Y11 (fiber direction)	30.3
	Y33 (electrode direction)	15.9
Shear modulus [GPa]	G12	5.5
Piezoelectric constant [pC/N or pm/V]	d_31_	−0.017
Poisson’s ratio	$v_{12}$	0.31
	$v_{21}$	0.16
Density [g/${cm}^{3}$]	ρ	0.544

**Table 2:** Vibration ratio of the AVC and passive gel.

Frequency [Hz]	Vibration Ratio [V/V]	
	AVC	Passive Gel
1	0.41	0.24
10	0.11	0.3
20	0.06	0.3
30	0.04	0.27
40	0.02	0.28
50	0.02	0.27

**Table 3:** Vibration ratio of the AVC and references.

Vibration Ratio [V/V]			
**AVC**	[32]	[34]	[35]
0.11	0.3	0.22	0.37 *

*: Vibration ratio [m.s^−2^/m.s^−2^].

## Data Availability

Data are contained within the article.
